# Listeriosis: Characteristics, Occurrence in Domestic Animals, Public Health Significance, Surveillance and Control

**DOI:** 10.3390/microorganisms12102055

**Published:** 2024-10-12

**Authors:** Ana Končurat, Tomislav Sukalić

**Affiliations:** Animal Disease Diagnostics Laboratory, Regional Department Križevci, Croatian Veterinary Institute, 48260 Križevci, Croatia; koncurat.vzk@veinst.hr

**Keywords:** *Listeria*, zoonosis, animals, foodborne pathogens, antimicrobial resistance, monitoring

## Abstract

Listeriosis is a dangerous zoonosis caused by bacteria of the genus *Listeria*, with *Listeria monocytogenes* (LM) being the most pathogenic species. *Listeria monocytogenes* has been detected in various animal species and in humans, and its ability to evolve from an environmental saprophyte to a powerful intracellular pathogen is driven by the invasion mechanisms and virulence factors that enable cell invasion, replication and cell-to-cell spread. Key regulatory systems, including positive regulatory factor A (PrfA) and the stress-responsive sigma factor σ^B^, control the expression of virulence genes and facilitate invasion of host cells. Listeriosis poses a significant threat to cattle, sheep and goat herds, leading to abortions, septicemia and meningoencephalitis, and ruminants are important reservoirs for *Listeria*, facilitating transmission to humans. Other *Listeria* species such as *Listeria ivanovii* and *Listeria innocua* can also cause disease in ruminants. Resilience of LM in food processing environments makes it an important foodborne pathogen that is frequently transmitted through contaminated meat and dairy products, with contamination often occurring along the food production chain. In humans, listeriosis primarily affects immunocompromised individuals, pregnant women and the elderly and leads to severe conditions, such as meningitis, septicemia and spontaneous abortion. Possible treatment requires antibiotics that penetrate the blood–brain barrier. Despite the relatively low antimicrobial resistance, multidrug-resistant LM strains have been detected in animals, food and the environment. Controlling and monitoring the disease at the herd level, along with adopting a One Health approach, are crucial to protect human and animal health and to minimize the potential negative impacts on the environment.

## 1. *Listeria* and Listeriosis

Listeriosis is a dangerous zoonosis caused by bacteria from the genus *Listeria*. These bacteria are small, Gram-positive, rod-shaped, catalase-positive, oxidase-negative, facultatively anaerobic, and non-sporulating. They are facultative intracellular pathogens, with the genus comprising 27 species divided in *Listeria sensu lato* and *Listeria sensu stricto* clades. By 2020, six *Listeria sensu stricto* species were known—*Listeria monocytogenes* (LM), *Listeria innocua*, *Listeria ivanovii*, *Listeria seeligeri*, *Listeria marthii* and *Listeria welshimeri*. Three new *sensu stricto* species were reported in 2021—*Listeria farberi* sp. nov., *Listeria immobilis* sp. nov. and *Listeria cossartiae* sp. nov.—which proved to be nonpathogenic [1], and by 2022 *Listeria swaminathanii* [2]. The most pathogenic species in the genus is LM, which can be divided into four evolutionary lineages (I, II, III, and IV), 13 serotypes grouped into 6 genoserogroups, and numerous clonal complexes (CCs). Lineage I (serotypes 4b and 1/2b) and lineage II (serotypes 1/2a and 1/2c) represent the majority of clinical isolates [3,4,5,6,7,8] and the majority of isolates from retail food samples as well [9]. The prevalence of serotype 1/2a is up to 61.1% [10]. Several CCs from lineage I of serotype 4b (including CC1, CC2, CC4 and CC6) are strongly associated with clinical cases in humans and have demonstrated hypervirulence [11]. Some strains of lineage I, such as CC1 and CC218, show a match in virulence profiles; however, in vitro studies on LM have shown that strains belonging to the same CC and possessing the same virulence genes can exhibit significant differences in adhesion and invasion abilities [12]. The most common hypervirulent strains, which are represented in clinical isolates from major outbreaks, appear to be distributed globally [13,14], geographically, and temporally, but the prevalence is uneven worldwide between cases of foodborne listeriosis and clinical cases. The most prevalent lineages in the Western world differ from those in Eastern Asia, suggesting that dietary habits and local specifics influence the geographical diversity of LM [15]. It is still not clear why the prevalence of certain CCs is higher in some countries (e.g., CC7 in Norway compared to most European countries), as no major differences were seen in virulence and stress response determinants when comparing those isolates [16].

Environmental pathogens like LM survive and replicate in the external environment but retain the ability to infect hosts. LM survives as a saprophyte in the soil but is capable of causing serious invasive disease in various animal species and humans. So far, the isolation of LM has been reported in humans, 42 species of domestic and wild mammals, 29 species of birds and in fish, crustaceans, frogs, snails, ants, ticks and flies [17]. It grows over a wide pH range and can multiply at refrigerator temperatures, posing a significant problem for the food industry. In *Listeria*, RNA-mediated regulation is triggered in response to changing environmental conditions, and recent findings indicate that non-coding RNAs (ncRNAs) regulate a variety of processes in this bacterium and contribute significantly to virulence and stress adaptation [18]. Low temperatures induce enzymes like RNA helicase, promoting activity and replication, and biofilm production enhances its ability to survive under adverse conditions [19]. Among the LM isolates, 3.5% demonstrate strong biofilm formation, while 38.5% show moderate biofilm production. A significant association was found between higher levels of biofilm production and the presence of the Stress Survival Islet 1, the *arsD* stress gene and the truncated inlA protein [20]. Biofilm formation in LM increases its virulence, adhesion and invasion of host cells, and drug resistance is significantly increased after biofilm formation [21].

## 2. Invasion Mechanisms and Virulence Factors of *Listeria*

**Figure 1:**
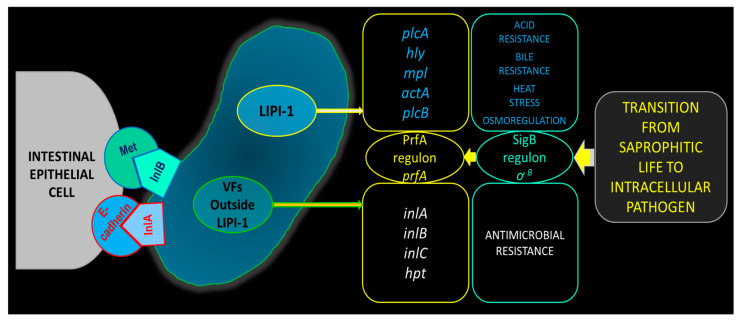
The critical virulence genes/virulence factors of *Listeria monocitogenes* facilitate its transformation from a saprophyte to a potent pathogen, allowing it to efficiently invade host cells. Abbreviations: LIPI-1—*Listeria* pathogenicity island 1; PrfA—positive regulatory factor A; SigB—Stress-responsive sigma factor σ^B^; plcA—phospholipase A; plcB—phospholipase B; actA—actin assembly-inducing protein; hly—listeriolysin O; mpl—metalloprotease; inlA—Internalin A; inlB—internalin B; inlC—Internalin C; hpt—hexose phosphate transporter.

*Listeria* secretes virulence factors (VFs) that promote cell invasion, bacterial replication and cell-to-cell spread, and the ability to bypass host barriers, escape the host immune system and survive depend on the expression of virulence genes (VGs) [22]. Key VGs/VFs involved in the pathogenesis of LM and their functions are listed in Table 1. VGs are located within both the core and accessory genomes, and pathogenicity islands (PAIs) contain clusters of these virulence genes. Four *Listeria* pathogenicity islands (LIPIs) have been identified in LM, of which LIPI-1 and LIPI-3 contain genes related to the infectious life cycle and survival in the food processing environment [23]. Among these, LIPI-1 includes a set of six genes (*hly*, *mpl*, *actA*, *plcA*, *plcB* and *prfA*) that are pivotal for virulence, each contributing uniquely to *Listeria* pathogenesis [24,25]. *Listeria* has highly sensitive protective and regulatory mechanisms, such as the stress-responsive sigma factor (σ^B^), a transcription factor that regulates the expression of genes responsible for bacterial survival under changing environmental conditions (stress response) [26,27], and the PrfA (positive regulatory factor A) system, which enables the transition from environmental saprophyte to intracellular pathogen [28]. σ^B^ modulates PrfA activity through direct transcriptional activation from the *prfA*P2 promoter and through indirect posttranscriptional repression under some environmental conditions. *prfA* expression is activated through an autoregulatory mechanism and by the branched chain amino acid sensor CodY; *prfA* translation is repressed by the ncRNA SreA [29]. PrfA induces the transcription of virulence factors located on the LIPI-1 (*hly*, *mpl*, *actA*, *plcA* and *plcB*), and additionally virulence factors outside LIPI-1 (e.g., *inlA*, *inlB*, *inlC* and *hpt*) that altogether are essential for the intracellular infection cycle [11,30,31,32] (Figure 1). The temperature influences the production of virulence factors because the secondary structure of untranslated *prfA*-mRNA is temperature-dependent [33,34]. At 30 °C, the sequence is blocked, preventing ribosomes from binding and translating the mRNA. However, at 37 °C, the secondary structure changes, allowing for the translation of *prfA*-mRNA and the synthesis of PrfA [35,36].

**Figure 2:**
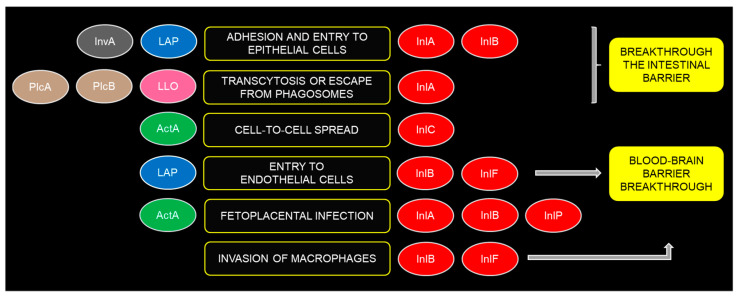
Virulence factors involved in *Listeria monocytogenes* adhesion and cell and tissue invasion. Abbreviations: InvA—*Listeria*-mucin-binding invasin A; LAP—*Listeria* adhesion protein; LLO—Listeriolysin O; plcA—phospholipase A; plcB—phospholipase B; actA—actin assembly-inducing protein; inlA—internalin A; inlB—internalin B; inlC—internalin C; inlF—internalin F; inlP—internalin P.

Additionally, internalins, another key group of virulence proteins, are essential for pathogenesis, with 11 internalin genes identified in *Listeria*. Internalins are critical for adhesion to and invasion of host nonphagocytic cells [39,40] and allow LM to bind to epithelial cells of the digestive tract through the host protein E-cadherin (InlA) [19] or through Met, gC1QR, and proteoglycans (InlB) [41], facilitating cellular entry (Figure 1). Five structurally related internalin proteins (InlA, InlB, InlC, InlF and InlP) play significant roles in LM pathogenesis. InlA enables transcytosis, InlB facilitates cellular entry, and InlC promotes cell-to-cell spread. InlA-mediated transcytosis allows for crossing the intestinal barrier, while InlF enables entry into endothelial cells to breach the blood–brain barrier (BBB). InlB also facilitates BBB crossing by prolonging the lifespan of infected monocytes, which then act as “Trojan horses” to transport bacteria to the brain. InlA, InlB and InlP contribute to fetoplacental infection [42] (Figure 2). LM can also produce chitinase (ChiA), the role of which was initially unclear as mammals do not synthesize chitin. However, it was revealed that ChiA has a specific functional role, enabling bacterial replication in target organs within the first 48 h of infection by inhibiting inducible nitric oxide synthase (iNOS), a critical cellular signaling molecule, thereby modulating the host’s innate immunity [43].

The expression of each disease symptom depends on the VFs present in *Listeria*, the host’s immune status, and the bacterial load ingested. Internalins (InlA and InlB), invasins (invasin A, LAP—*Listeria* adhesion protein), and surface adhesion proteins (InlP1, InlP4) are responsible for binding to epithelial cells, while internalin C and actin-inducing protein (ActA) are involved in cell-to-cell spread [37]. InlB is identified as the ligand of secreted LAP on the LM cell surface, and LAP-InlB interplay promotes LM systemic spread, including brain invasion [38]. In eukaryotic cells, LM is at first transiently trapped in a primary vacuole from which it escapes prior to phagolysosomal fusion through membrane pores formed by listeriolysin O (LLO) and is further facilitated by the following two phospholipases: PlcA and PlcB [11]. The actin assembly-inducing protein (ActA) promotes intracellular bacterial motility and facilitates cell-to-cell spread by polymerizing actin from the host cytoskeleton [11,44,45,46] (Figure 2). Rapid switching of gene expression pathways and protein synthesis is a strategy for *Listeria* to adapt quickly to the host’s dynamic environment [47].

LM has a special tropism for the pregnant uterus and is able to cross the placenta and BBB and penetrate the fetus and central nervous system (CNS) [48]. Its ability to invade and replicate within epithelial cells and caruncles is linked to resistance to lysozyme, also suggesting potential survival in challenging environments like those in abomasum [49]. However, LM, like other bacteria, forms metabolic products within cells that the host’s immune system can recognize. One bacterial metabolite is cyclic dimeric adenosine monophosphate (c-di-AMP), a cyclic dinucleotide that controls growth, cell wall homeostasis, and virulence. Its secretion into the cytosol enables recognition by the host protein STING (stimulator of interferon genes), triggering type I interferon production and the interferon response [50].

## 3. Listeriosis in Domestic Animals, Prevalence in Ruminants and *Listeria* Reservoirs for Humans

Manifestations of infection in mammals include abortions and perinatal mortality. In adult ruminants, encephalitis or meningoencephalitis occurs, while neonatal ruminants and monogastric animals primarily develop septicemia (Table 2). Ruminants play a crucial role in the persistence and transmission of LM through a continuous oral–fecal cycle, making farms a significant reservoir of this microorganism and a key point in the transmission pathway from animals to humans [51]. They can be asymptomatic carriers or develop clinical manifestations, such as neurological or fetal infections, similar to humans [25]. Research indicates that cattle are routinely exposed to hypervirulent LM CCs. Lineage I hypervirulent clonal complexes (CC1, CC2, CC4 and CC6) have been significantly associated with fetomaternal and, notably, CNS infections, suggesting a heightened ability to breach placental and neural barriers. [52,53]. The most frequently associated CCs with clinical cases in cattle and sheep is CC1, predominantly linked to rhombencephalitis and abortions but also found in environmental samples. Abortions are often associated with CC37 and CC6 [54].

The reported prevalence of *Listeria* on 27 cattle farms in Latvia was 58.9%, while for LM it was 11%. The highest prevalence of LM was found in the environmental soil samples taken near the manure storage area (93%), and the predominant LM CCs were CC37 (30%), CC11 (20%) and CC18 (17%), all serogroup IIa [55]. In samples from dairy farms in Portugal, LM was found in feces (12.5%), feed (12.0%) and water (8.3%) [51]. A study conducted in Korea [56] on LM isolates from domestic ruminants showed the highest prevalence of CC1, CC365 and CC91 and serotypes 4b, 1/2a and 1/2b. This study also revealed that atypical clones can cause disease with clinical manifestations and confirmed histopathological changes. In a large study conducted in Spain [57], which involved 343 herds of healthy ruminants and pigs (120 sheep flocks, 124 beef cattle herds, 82 dairy cattle herds and 17 pig herds), LM was found in 46.3% of dairy cattle herds, 30.6% of beef cattle herds and 14.2% of sheep flocks, while no LM was isolated from pig herds. The most common serovar was 4b (84.2%), followed by 1/2a (13.2%), indicating that cattle are potentially significant reservoirs of LM.

**Table 2:** The most common forms of listeriosis caused by *Listeria monocytogenes* in different hosts and possible infections with *Listeria ivanovii* and *Listeria innocua*.

Host/Disease Form		Meningitis– Encephalitis	Septicemia	Abortion	Kerato- Conjunctivitis	References
Domestic mammals	Adult ruminants	*L. monocytogenes*		*L. monocytogenes* *L. ivanovii ^(SR)^*	*L. monocytogenes*	[25,56,58,59,60]
	Neonatal ruminants		*L. monocytogenes*			[61,62]
	Monogastric		*L. monocytogenes*			[61]
Birds	Domestic/wild		*L. monocytogenes* *L. innocua ^(P)^*			[63,64]
Humans	Neonates	*L. monocytogenes*	*L. monocytogenes*			[52,65,66]
	Pregnant women			*L. monocytogenes* *L. ivanovii ^(P)^*		[67,68]
	Immunocompromised		*L. monocytogenes* *L. ivanovii ^(P)^*			[69,70,71]
	Elderly	*L. monocytogenes*	*L. monocytogenes*			[52,69,70,71]

(SR)—small ruminants; (P)—potentially pathogen.

The reported LM prevalence among cattle abortion cases was 16.1% [72], predominantly occurring in the third (64.4%) and second (33.3%) trimesters, mostly in spring, affecting heifers and cows up to 4 years old. Key virulence factors, such as the prfA-dependent virulence cluster and *inlA* and *inlB*, were found in all LM isolates. In an outbreak of abortions among 23 heifers [73], 19 were confirmed to have LM, with eight strains belonging to lineage I and 11 to lineage III, directly linked to water and silage contaminated with lineage III LM. Research on sheep abortions in Australia showed that in cases with an etiological diagnosis, 26% were due to *Listeria*, including 14% *Listeria* spp., 10.7% *L. ivanovii*, 1% LM and 0.3% *L. innocua* [58]. It is determined that 4.66% of total cattle abortions in Tunisia were caused by LM [74]. A study conducted in Croatia on 373 samples from domestic ruminant abortion cases resulted in 54 isolated strains of *Listeria*, accounting for 14.47% positive samples, with LM isolated in 38 cases (10.18%). *L. innocua*, facultatively pathogenic for ruminants, was found in 2.68% of cases and other nonpathogenic *Listeria* species in 1.6%, while *L. ivanovii* was not detected. LM was isolated in 6.55% of cattle cases and in 24.61% of small ruminant cases [59].

Improperly prepared and spoiled silage is considered the primary source of pathogenic *Listeria* for ruminants. LM was isolated almost five times more frequently from silage samples with a pH above 4.5 than from those with a lower pH [75]. *Listeria* keratoconjunctivitis and uveitis (“silage eye”) is a specific disease entity in cattle, resulting from direct LM entry into the eye. One of the primary defense mechanisms in tears is lysozyme (concentrations of about 580 µg/mL in cattle and up to 2 mg/mL in humans), to which LM can be naturally resistant, with some strains capable of modifying its peptidoglycan (PG) structure to evade enzymatic degradation [60].

In addition to LM, which can cause various disease forms in different animal species, *L. ivanovii* is pathogenic, causing abortions in sheep and cattle. Although rare, *L. innocua* can also be pathogenic for sheep [48]. *L. ivanovii* shows a host tropism toward small ruminants and rodents, with much lower virulence for humans compared to LM. It has been isolated from the placenta, brain, liver, lungs and abomasal content of ruminants. Sources of *L. ivanovii* include various food products, bacteria have capability of surviving in food processing facilities, and so far were isolated from a wide range of animal and plant matrices, including raw milk (cow, sheep and goat), dairy products (butter and cheese), raw, cured, and frozen beef, pork, lamb, goat meat, rabbit meat, chicken, turkey, canned fish, and various vegetables like lettuce, leafy greens, tomatoes, cabbage, beans, potatoes and rice [76].

*L. innocua* is generally considered nonpathogenic due to the lack of major virulence genes (*prfA*, *plcA*, *hly*, *mpl*, *actA* and *plcB*), which are located in the LIPI-1 pathogenicity island in LM. However, LIPI-4 pathogenicity island which is characteristic for certain hypervirulent strains (e.g., CC4) was found in *L. innocua* [13]. Moreover, a recent study identified the LIPI-3 pathogenicity island, which encodes listeriolysin-S, in 20% of *L. innocua* isolates [77] and various virulence genes, such as *hly*, *plcA* and *inlC*, were found in atypical *L. innocua* strains isolated from pig slaughterhouses and meat shops [78]. The genetic diversity of *L. innocua* clones, widely spread in farm environments, indicates that certain isolates have significant pathogenic potential for cattle. The incidence of *L. innocua* in cattle and sheep farm and slaughtering environments is more common and significantly higher (9.7%, 508/5214) than that of LM (1.8%, 94/5214) [79]. In the study conducted on 110 isolates of *L. innocua*, 14 different sequence types (STs) were detected, carrying 13 resistance genes and 23 virulence genes, of which 13 (*clpC*, *clpE*, *clpP*, *hbp1*, *svpA*, *hbp2*, *iap/cwhA*, *lap*, *lpeA*, *lplA1*, *lspA*, *oatA*, *pdgA* and *prsA2*) were found in all 110 isolates of *L. innocua* [80].

Domestic and wild birds also serve as *Listeria* reservoirs. Generally, young birds are more susceptible to infection and more likely to develop clinical signs than are older birds. Although clinical listeriosis in birds is rare, outbreaks have been reported after stressful events [63]. A study conducted on gull populations at a landfill in Zagreb (Croatia), reports *L. innocua* in 14.4% of samples and LM in 11.3% of samples from 390 gulls [64]. In poultry, ubiquitous hematophagous ectoparasite infesting egg-laying hens, *Dermanyssus gallinae*, can carry LM [81].

## 4. *Listeria* and Public Health Significance

### 4.1. Contamination of Animal-Origin Products

LM is one of the most significant foodborne pathogens because of its widespread presence and resistance to adverse conditions, with cattle and sheep as natural hosts, which can transmit LM to related meat and dairy products. The analysis of different molecular and genetic characteristics of *Listeria* by pulsed-field gel electrophoresis (PFGE) and multilocus sequence typing (MLST) suggest that LM and *L. innocua* are gradually transmitted from the farm and slaughter environment to the final products along the slaughtering chain [79]. A study conducted on 1148 food and environmental samples in Croatia found LM in 28 (2.44%) samples [82]. From samples of raw pork and ready-to-eat foods in Italy, 8.7% were positive for LM, but the contamination flow of LM has a low occurrence in slaughterhouses and increased throughout the processing chain with trimming area as the most contaminated zone [83]. One Chinese study showed that a packaging room had the most contamination (48.3%), with a peak occurrence of 76.5% in processing environments [84]. Another study on samples from slaughterhouses and pork processing facilities showed 243 out of 2496 samples (9.74%) positive for LM, with persistent strains more frequently detected in food samples, which are linked to clinical cases in humans [85]. Persistent strains are genetically similar and isolated from the same locations over intervals of six months or more. Their presence has also been confirmed in food businesses and restaurants [86]. LM was found in 26.66% of the beef samples analyzed from butcher shops at open markets in Brazil, indicating the unhygienic and unsanitary conditions at the sampling site [87]. Dairy farms are highlighted as a reservoir for hypervirulent LM [88]. It is important to note that *Listeria* excretion in milk post-infection is intermittent and prolonged, and *Listeria* can survive the pasteurization process. Fresh cheese is an ideal substrate for microorganisms, and this is corroborated by the 3.3% LM-positive fresh cheese samples found in local markets, while *L. innocua* was isolated from 10% of samples [89]. To inhibit the growth of LM in cheese, bacteriocinogenic LAB (lactic acid bacteria) strains contained in primary, adjunct or protective cultures are acceptable in cheese production [90].

### 4.2. Significance for Human Health

In humans, LM can cause disease in the elderly, children, immunocompromised individuals (meningitis, encephalitis, altered mental status and sepsis) and pregnant women (spontaneous abortion) or cause fever and limited gastroenteritis in healthy adults (Table 2). According to an FAO/WHO JEMRA MRA38 report [69], there are three main subpopulations, as follows: (1) less susceptible subpopulation—population under 65 years with no known risk factors for listeriosis or comorbidities; (2) susceptible subpopulation—pregnant women and their newborns, as well as adults aged 65 or older; and (3) very susceptible subpopulation—people with weakened immune systems, such as individuals with HIV, cancer patients and organ-transplant patients. Based on susceptibility, consideration should be given to a dose–response (DR) model for these three main subpopulations. The DR model represents the relationship between the number of ingested pathogenic organisms and the probability of infection or illness, and the DR relationship for human listeriosis is affected by the following three aspects: (1) the food matrix, (2) host susceptibility, and (3) pathogen characteristics/virulence. The estimated relative risks (RRs) for listeriosis after ingestion of 1000 bacteria of “less virulent” versus “virulent” strains, ranged from 21.6 to 24.1 depending on the subgroup. These relatively low RRs compared to the RRs associated with comorbidities suggest that the influence of comorbidities on the occurrence of invasive listeriosis at a given exposure is much more important than the influence of strain virulence [91].

In the United States, approximately 1600 people become ill annually, with 260 infections resulting in death, making listeriosis the third leading cause of death among foodborne illnesses [19]. Out of 13 LM serotypes, only serotypes 1/2a, 1/2b, 1/2c and 4b cause clinical disease in humans, with pregnant women most commonly infected with serotype 4b. Gestational age at the time of infection significantly influences prognosis; if diagnosed in the first trimester, 65% of pregnant women will miscarry, whereas in the second and third trimesters, this occurs in 26% of cases [67]. LM infection was reported in 4 out of 109 (3.66%) women with spontaneous abortion [68]. After an incubation period of about three weeks, pregnant women typically experience flu-like symptoms, but *Listeria* can reach the uterus, leading to fetal death and miscarriage or severe listeriosis in the newborn [65]. Cases of listerial meningitis in the postpartum period have also been documented, which is considered a critical period due to changes in immune response and increased risk of infections [66]. *L. ivanovii* can occasionally cause infections in humans, resulting in bacteremia in immunocompromised individuals and stillbirths and abortions in pregnant women [76].

Although listeriosis is relatively rare in humans, it often has a severe disease course, frequent hospitalization, and high mortality rates. In 2017, more than 2400 cases were reported in European Union (EU) countries [92]. According to the EU One Health zoonoses report for 2021, LM infections rank fifth among reported zoonoses, behind salmonellosis, *Campylobacter* spp. infections, yersiniosis, and Shiga-toxin producing *Escherichia coli* (STEC) infections. However, LM infections, along with those caused by the West Nile virus, were the most severe, with the highest number of hospitalizations and mortality rates [93]. The European Centre for Disease Prevention and Control (ECDC) reports the highest incidence of illness in individuals over 64 years old, with the most cases in Germany, France, and Spain [94]. Over the past five years, the number of reported cases in the EU ranged from 1931 to 2652 annually [65]. Increase in severe food-borne infections, including listeriosis is reported in the EU in 2022, and the numbers of reported foodborne outbreaks and cases, hospitalizations and deaths were higher in 2022 than in 2021. The number of deaths from outbreaks was the highest ever reported in the EU in the last 10 years, mainly caused by *L. monocytogenes* [95,96], and between 2012 and 2024 a prolonged outbreak of 73 cases of LM ST173 infections (“My2”-cluster) has been ongoing in Belgium (5), Czechia (1), Germany (39), Finland (2), Italy (1), the Netherlands (20) and the United Kingdom (5). Fourteen deaths have been recorded as associated with this outbreak, and most of the patients for whom information was available from interviews had consumed various fish products [97]. The data suggest frequent detection of LM in ready-to-eat (RTE) fish products [98], and the number of cases connected with salmon products and RTE fish products has sharply increased after 2021. In the period from 1996 to 2018, the usual food vehicles traditionally associated with the LM transmission were unpasteurized milk and dairy products, soft cheese varieties, cooked, ready-to-eat sausages and sliced meats, refrigerated seafood, smoked seafood, pâtés and meat spreads [99]. The most recent case was reported in March 2024 in Denmark, indicating an ongoing risk of further infections. Of 20 listeriosis cases, five have died, indicating high severity of infection particularly among elderly people with underlying chronic conditions [70,71].

## 5. Treatment, Antimicrobial Resistance, Disease Surveillance and Control Measures

Among domestic animals, listeriosis poses a significant risk primarily to cattle, sheep and goat herds, with swine being less affected and horses and poultry rarely affected. The greatest risk lies in frequent abortions, significant losses of offspring and deaths due to septicemia. Abortions typically occur in the last third of gestation, leading to major economic losses as the pathogen spreads into the environment through aborted fetuses, placentas and amniotic fluid in pastures. The rapid spread within a herd due to a short incubation period and the potential for up to 20% of animals to abort, along with meningoencephalitis affecting 10–30% of animals in some cases, are alarming factors [61], and each case of listeriosis in cattle and small ruminant herds results in additional losses.

### 5.1. Treatment Options and Antimicrobial Resistance

The treatment of listeriosis in animals is possible but requires antimicrobial agents that can penetrate intracellularly and cross the blood–brain barrier. High doses are needed to achieve the minimal bactericidal concentration (MBC) in the brain. It usually involves β-lactam antibiotics, tetracyclines, erythromycin, gentamicin and sulfamethoxazole/trimethoprim. The standard treatment for listeriosis in humans involves β-lactams, particularly aminopenicillins like ampicillin or amoxicillin, typically combined with an aminoglycoside, most often gentamicin, to enhance the synergistic bactericidal effect. For patients allergic to β-lactams, co-trimoxazole is commonly used, as sulfamethoxazole boosts the bactericidal activity of trimethoprim, despite LM isolates often showing resistance to sulfonamides. The use of vancomycin, fluoroquinolones, rifampicin and linezolid has also been reported [48,100,101,102], and during the last decade no increased resistance of LM to antimicrobials was observed [103].

Antimicrobial susceptibility tests showed that >90% of isolates were susceptible to penicillin, ampicillin, erythromycin, ciprofloxacin, sulfamethoxazole–trimetoprim, vancomycine, chloramphenicol and gentamycin, indicating the antibiotic treatment might be still efficient for most of the LM strains [9]. Although *Listeria* resistance is still low, multiresistant strains have been isolated from diseased humans, animals, food and the environment [48]. LM isolates from bulk-tank bovine milk in Greece were all characterized as multidrug resistant, with resistance to penicillin and clindamycin being a common feature [104]. Also, all of the LM sequence types from a South African study carried four intrinsic/natural resistance genes, *fosX*, *lin*, *norB* and *mprF*, conferring resistance to fosfomycin, lincosamide, quinolones, and cationic peptides [105]. Extensive research in the EU, USA, and Asia shows the presence of resistance genes like *fosX*, *lin*, *abc-f* and *tet(M)* in LM, indicating potential resistance to quinolones, lincomycin, cephalosporins and oxytetracycline [106]. Alternative therapeutic options, such as bacteriophages, egg yolk antibodies, cytokines, herbs, essential oils and probiotics, could pave the way for the development of valuable and effective treatment modules to combat listeriosis [107].

### 5.2. Surveillance and Control Measures

Despite regulated controls of food and environmental samples from food processing facilities, the resilience, persistence and spread of *Listeria* within herds and into the food production chain, necessitate herd-level disease monitoring as the “first line of defense”.

Agricultural land use can raise health concerns, as soil can transfer human pathogens to crops, farm animals and food. LM is naturally found in soil and its persistence is due to intrinsic factors like transport systems and transcriptional regulators. However, its survival is also influenced by external factors such as soil conditions and the biotic environment [108]. Factors beyond time also affect the presence of LM in soil. Environmental factors like weather and soil type; management practices like manure application; and the presence of other microbes (such as *E. coli* and other pathogens) were also linked to LM contamination [109]. Soil survival did not depend on the origin of strains or their phylogenetic position, and a greater number of genes specifically associated with good soil survival were found in lineage II strains than in lineage I strains. Soil fitness was mainly associated with variations in genes encoding membrane proteins, transcriptional regulators and stress resistance genes in both lineages, as well as genes encoding proteins related to motility and genes in the category “phage-related genes” [110]. *LM* is commonly found in silage, haylage, grazing pastures, farmyards and water. Feces of wild animals, including gulls and crows, have also been described as important vectors of the pathogen for farm animal contamination, as well as animal bedding, especially when animals are housed during indoor months [111].

Monitoring of cattle, sheep and goat herds, as well as swine production, can significantly reduce *Listeria* entry into food processing facilities (slaughterhouses, meat processing plants and dairies). Although listeriosis is listed in EU legislation [112] and meets the criteria of the Animal Health Act for inclusion in the National Lists of Diseases, current regulations in European countries mostly do not include specific measures for listeriosis control and surveillance on herd level. Moreover, in 2024, LM was removed from the World Organization for Animal Health (WOAH) Terrestrial manual [113]. But the low number of reported cases of listeriosis is in strong contrast to the results of surveillance studies. For instance, active surveillance study in Switzerland revealed that listeriosis is the most common CNS disease in the small ruminant population and that its prevalence is highly underestimated [114].

Considering the damage caused by the disease, surveillance program measures should include, at least, the following:

- Mandatory testing of abortion samples, ensuring all aborted material from livestock is tested for listeriosis;
- Mandatory testing for listeriosis in small ruminants and cattle carcasses if CNS symptoms are present;
- Implementing mandatory preventive rodent-control and bird-control measures on farms and disinfection measures, following disease confirmation;
- Additionally, educational programs for animal owners and veterinarians should be planned to improve awareness and response to listeriosis.

By implementing these measures, it is possible to significantly mitigate the impact of listeriosis on livestock and reduce its entry into the food production chain, thereby protecting both animals and public health.

## 6. Future Research—Need for One Health Approach

Listeriosis is a disease that relentlessly demonstrates how human health is connected to the health of animals and our shared environment. As part of the One Health concept, which recognizes the interconnectedness of human, animal and environmental health, listeriosis is a good example of how diseases can affect multiple species. There are several One Health issues associated with listeriosis, such as its zoonotic nature, food safety and environmental impact through soil and water contamination [62]. Despite the food safety criteria for listeriosis introduced by EU Regulation (EC) No 2073/2005 [115], there is an increasing trend of listeriosis in humans and the presence of LM in RTE foods represents one of the major challenges for the food industry [116]. The fact that a high percentage of all LM isolates recovered from the milk and dairy products harbored one or more resistance genes encoding resistance against various antibiotics [117] and aforementioned multiresistant strains isolated from humans, animals, food and the environment, necessitate the recognition of the One Health approach to ensure food safety and contain the spread of antimicrobial resistance in foods. As *Listeria* has been found in the pre-harvest and on-farm environments, environmental surveys are an important tool to improve our understanding of the presence and prevalence of *Listeria* in irrigation water sources and their surroundings [118]. It is recognized that an interdisciplinary, integrative and international approach is essential to address the existing and emerging threats of zoonotic diseases and antimicrobial resistance [119]. In recent years, a large dataset of LM genomes from diverse ecological niches and phenotypic data from a panel of LM strains have been collected [120], contributing to a better understanding of the LM population’s structure and evolutionary history, facilitating the detection of emerging LM clones and identifying genetic traits related to LM adaptation to specific ecological niches.

Continued research has significantly improved our knowledge of *Listeria* and listeriosis, and future research in this field should focus on LM mechanisms of adaptation to stress in different ecological niches and mechanisms of immune tolerance by the host. It is necessary to conduct systematic sampling in different interrelated systems (soil, water, farm environment, animals, food processing plants, foods, food serving facilities, humans and health institutions) in order to compare research results.

## 7. Conclusions

LM is highly adaptable and capable of causing severe infections in humans and animals. The bacterium is particularly dangerous because of its ability to survive in various environmental conditions, including low temperatures, and its capacity to form biofilms that protect it from adverse conditions. The pathogenicity of LM is largely determined by its VFs, which facilitate its invasion of host cells, crossing of the blood–brain barrier and placenta, and its ability to spread from cell to cell. The virulence of LM varies among its different lineages and clonal complexes, with some strains being hypervirulent. Despite possessing similar virulence genes, strains can exhibit varying levels of pathogenicity, indicating that other factors, such as environmental conditions and host interactions, play a crucial role in infection outcomes.

Listeriosis can cause serious economic losses in livestock production, primarily through abortions and the death of young animals, as well as fatalities due to septicemia. Consequently, this can negatively impact the food industry because of the contamination of animal-derived products. The occurrence of listeriosis can have a significant negative impact on the environment, as the remains of aborted fetuses, placentas and amniotic fluid in the environment, especially in animals kept on pasture, lead to the spread of the pathogen and contamination of the soil and pastures. The pathogens can survive in the soil and water and contaminate animal feed. Rodents, as disease reservoirs, and birds, as carriers, further spread the pathogen to distant herds, increasing the risk of disease spread. Listeriosis poses a significant threat to the health of both humans and animals.

Due to its zoonotic nature, the resistance of the causative agent to adverse conditions and the risk of transmission through the food chain, listeriosis certainly deserves more attention. Interconnections between humans, animals and the environment within the context of listeriosis require One Health approach in order to improve disease prevention, surveillance and control.

## Figures and Tables

**Table 1:** Key virulence genes/virulence factors involved in pathogenesis of Listeria monocytogenes and their functions.

Listeria Monocytogenes—Main Virulence Genes/Virulence Factors Involved in Pathogenesis				
Virulence Gene	Gene Product	Full Name	Function	References
*sigB*	SigB	General stress-responsive sigma factor σ^B^	Major stress response regulator, biofilm formation, resistance of biofilm-grown cells to the disinfectants	[27]
*prfA*	PrfA	Positive regulatory factor A	Intracellular survival and cell-to-cell spread, activation of PrfA-dependent genes on “PrfA island” or *Listeria* pathogenicity island 1 [LIPI-1]	[29]
*plcA*	PlcA	Phospholipase A	Mediate vacuolar escape	[11]
*plcB*	PlcB	Phospholipase B	Mediate vacuolar escape	[11]
*actA*	ActA	Actin assembly—inducing protein	Cell-to-cell spread	[37]
*hly*	LLO	Pore-forming toxin listeriolysin O	Escape from the phagosome (vacuole)	[29]
*mpl*	Mpl	Metalloprotease	PclB activation, vacuole escape, ActA processing and protrusion resolution	[31]
*inlA*	InlA	Internalin A	Adhesion and entry to enterocytes, receptor-mediated endocytosis	[11,37]
*inlB*	InlB	Internalin B	Adhesion and entry to enterocytes, ligand of LAP, brain colonization, receptor-mediated endocytosis	[11,37,38]
*inlC*	InlC	Internalin C	Cell invasion	[37]
*inlF*	InlF	Internalin F	Adhesion and invasion of macrophages, brain colonization	[37]
*inlP*	InlP	Internalin P	Placental colonization	[37]
*hpt*	Hpt	Hexose phosphate transporter	Sugar phosphate uptake	[32]
*lap*	LAP	*Listeria* adhesion protein	Adhesion and entry to enterocytes	[37]
*lmo1413*	InvA	*Listeria*-mucin-binding invasin A	Intestinal mucus penetration	[37]

## Data Availability

No new data were created or analyzed in this study.
